# A New Preclinical Decision Support System Based on PET Radiomics: A Preliminary Study on the Evaluation of an Innovative ^64^Cu-Labeled Chelator in Mouse Models

**DOI:** 10.3390/jimaging8040092

**Published:** 2022-03-30

**Authors:** Viviana Benfante, Alessandro Stefano, Albert Comelli, Paolo Giaccone, Francesco Paolo Cammarata, Selene Richiusa, Fabrizio Scopelliti, Marco Pometti, Milene Ficarra, Sebastiano Cosentino, Marcello Lunardon, Francesca Mastrotto, Alberto Andrighetto, Antonino Tuttolomondo, Rosalba Parenti, Massimo Ippolito, Giorgio Russo

**Affiliations:** 1 Institute of Molecular Bioimaging and Physiology, National Research Council (IBFM-CNR), 90015 Cefalù, Italy; viviana.benfante@ibfm.cnr.it (V.B.); alessandro.stefano@ibfm.cnr.it (A.S.); selene.richiusa@ibfm.cnr.it (S.R.); milene.ficarra@ibfm.cnr.it (M.F.); giorgio.russo@ibfm.cnr.it (G.R.); 2 Department of Health Promotion, Mother and Child Care, Internal Medicine and Medical Specialties, Molecular and Clinical Medicine, University of Palermo, 90127 Palermo, Italy; bruno.tuttolomondo@unipa.it; 3 Ri.MED Foundation, Via Bandiera 11, 90133 Palermo, Italy; acomelli@fondazionerimed.com (A.C.); pgiaccone@fondazionerimed.com (P.G.); 4 Nuclear Medicine Department, Cannizzaro Hospital, 95126 Catania, Italy; fabrizioscopelliti@gmail.com (F.S.); marco.pometti@gmail.com (M.P.); ianocose@hotmail.com (S.C.); centro_pet@ospedale-cannizzaro.it (M.I.); 5 Department of Physics and Astronomy, University of Padova, 35131 Padova, Italy; marcello.lunardon@unipd.it; 6 Department of Pharmaceutical and Pharamacological Sciences, University of Padova, 35131 Padova, Italy; francesca.mastrotto@unipd.it; 7 Legnaro National Laboratories, National Institute of Nuclear Physics, 35020 Legnaro, Italy; alberto.andrighetto@lnl.infn.it; 8 Department of Biomedical and Biotechnological Sciences, University of Catania, 95123 Catania, Italy; parenti@unict.it

**Keywords:** radiomics, micro-PET/CT, mouse imaging, atlas, ^64^Cu-labeled chelator

## Abstract

The ^64^Cu-labeled chelator was analyzed in vivo by positron emission tomography (PET) imaging to evaluate its biodistribution in a murine model at different acquisition times. For this purpose, nine 6-week-old female Balb/C nude strain mice underwent micro-PET imaging at three different time points after ^64^Cu-labeled chelator injection. Specifically, the mice were divided into group 1 (acquisition 1 h after [^64^Cu] chelator administration, n = 3 mice), group 2 (acquisition 4 h after [^64^Cu]chelator administration, n = 3 mice), and group 3 (acquisition 24 h after [^64^Cu] chelator administration, n = 3 mice). Successively, all PET studies were segmented by means of registration with a standard template space (3D whole-body Digimouse atlas), and 108 radiomics features were extracted from seven organs (namely, heart, bladder, stomach, liver, spleen, kidney, and lung) to investigate possible changes over time in [^64^Cu]chelator biodistribution. The one-way analysis of variance and post hoc Tukey Honestly Significant Difference test revealed that, while heart, stomach, spleen, kidney, and lung districts showed a very low percentage of radiomics features with significant variations (*p*-value < 0.05) among the three groups of mice, a large number of features (greater than 60% and 50%, respectively) that varied significantly between groups were observed in bladder and liver, indicating a different in vivo uptake of the ^64^Cu-labeled chelator over time. The proposed methodology may improve the method of calculating the [^64^Cu]chelator biodistribution and open the way towards a decision support system in the field of new radiopharmaceuticals used in preclinical imaging trials.

## 1. Introduction

Before moving into clinical trials, the use of animal models is essential. Nowadays, different types of animal models are used in preclinical stages of experimentation and a well-defined murine model, based on the goal that must be achieved, is used for each specific experimental stage. For example, murine toxicology studies are required for radiopharmaceutical dose characterization. In spite of that, pharmacological and toxicological studies can be translated into experimental studies on human primary tumors with the aim of selecting new therapeutic targets, starting from orthotopic mouse models of specific human tumors [1]. This type of orthotopic transplantation in a murine immunodeficiency model is the cause of the development of metastases mainly in organs such as liver, lungs, and bones. This last type of metastasis can generally also be induced using intravenous or intracardiac cell injection [2,3,4]. These assumptions justify the choice of our murine model in this study, since our next studies on a new radiopharmaceutical biodistribution on mice inoculated with tumor cells will be conducted on the same animal model used here.

**Figure 1:**
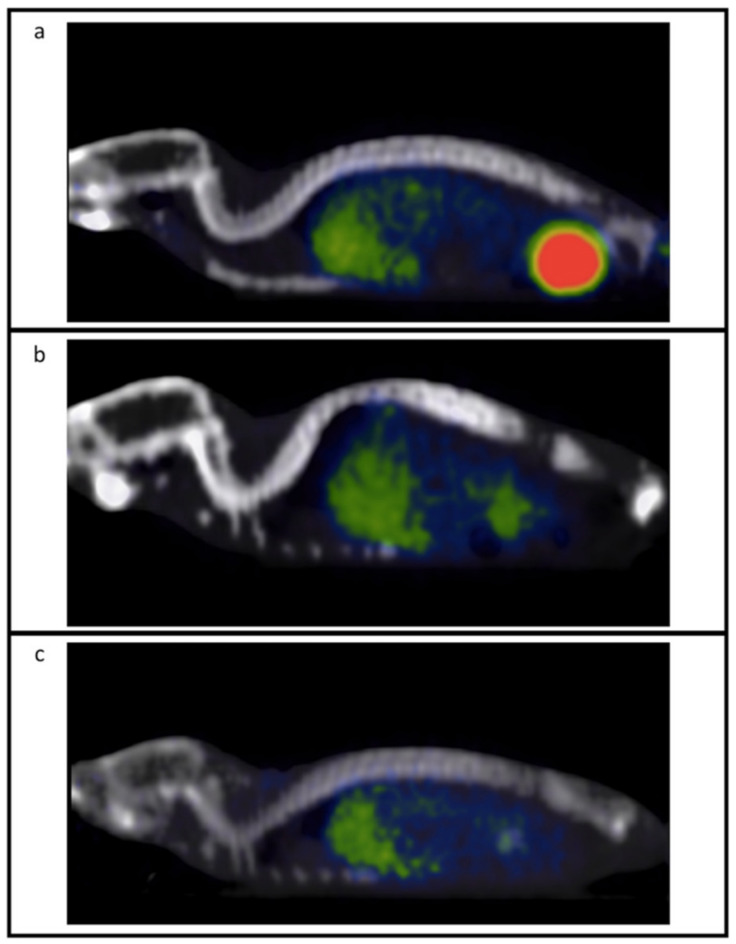
PET/CT images of three mice injected with the ^64^Cu-labeled chelator at the three acquisition times via micro-PET/CT (Albira Si micro-PET/CT). (**a**) Mouse scanned 1 h after administration of the radioactive compound; (**b**) mouse scanned 4 h after administration of the radioactive compound; (**c**) mouse scanned 24 h after administration of the radioactive compound.

In this context, molecular imaging plays a fundamental role, in particular with positron emission tomography (PET). Furthermore, dual-mode imaging, such as PET/computed tomography (CT) imaging, allows the integration of anatomical with physiological characteristics of the disease. Radio-metallic nuclides are the basis of nuclear medicine studies for diagnostic and therapeutic applications [5]. These nuclides can be contained in the structure of special kinds of drugs, namely, radiopharmaceuticals [5]. Radiopharmaceuticals used in diagnosis or therapy are characterized by different and specific types of nuclear profiles. Radionuclides used in the diagnostic field emit positrons (β+) or gamma rays (γ) such as Fluorine-18 (^18^F), Zirconium-89 (^89^Zr), and Gallium-68 (^68^Ga), which are among the most used in PET imaging. Radionuclides used in the therapeutic field emit alpha (α), beta (β−) particles, or Auger electrons. These kinds of particles act by inducing potential cytotoxicity on target tissues (such as tumor tissues) through a specific ionization process that causes damage to the Deoxyribonucleic Acid (DNA) of tumor cells. Generally, a diagnostic radiopharmaceutical is coupled to a therapeutic radiopharmaceutical. This underlies the concept of theranostics, according to which the radionuclides used in diagnosis and therapy are considered as a matched pair. An already-known example of theranostic Copper (Cu) radiopharmaceuticals is represented by the pair formed ^64^Cu/^67^Cu [5]. ^64^Cu is used in PET (diagnosis) and in radionuclide therapy thanks to its physical characteristics and its special decay profile (T_1/2_ =12.7 h; β+: 19%, β−: 38%), and therefore it is considered an excellent candidate in the theranostic approach [6]. ^64^Cu enables high-quality and -contrast PET images and guarantees reliable absorption and distribution, thanks to its long half-life. ^64^Cu can be linked to different targeting systems such as antibodies, peptides, proteins, and other biologically important small molecules [7], and it is very promising for tumor-targeted radiotherapy [8]. The ^64^Cu chelator considered in our study is DO2A2S, a derivative of DOTA extensively discussed in [9]. The advantage of using this new in vivo chelator is represented by the fact that ^64^Cu-labeled DOTA derivatives [10] are more stable in vivo than those based on ^64^Cu-labeled TETA [10]. In particular, DO2A2S is known to be capable of exploiting the 2+ and 1+ oxidation state of copper to coordinate, respectively, with the two carboxylated arms and the two sulfured arms [9]. Consequently, preclinical research is deeply involved in animal experimentation to validate the efficacy of new chelators for ^64^Cu. For this reason, the aim of this study is to present a new preclinical decision support system based on micro-PET/CT images to identify several mouse organs and to assess, through a radiomics analysis, the biodistribution of [^64^Cu] chelator at the three time points after the injection. Usually, radiomics studies aim to reflect the tumor pathophysiology and, potentially, the development of the disease through the implementation of a predictive model based on the extraction of relevant features from medical images and the use of statistical models, i.e., [11]. In this study, radiomics is used in an innovative way, i.e., with the aim of implementing an operator-independent tool to study the ^64^Cu-labeled tracer biodistribution in PET imaging over time. To achieve this goal, nine mice divided into group 1 (acquisition 1 h after [^64^Cu] chelator administration, n = 3), group 2 (acquisition 4 h after [^64^Cu] chelator administration, n = 3), and group 3 (acquisition 24 h after [^64^Cu] chelator administration, n = 3) were analyzed. Successively, radiomics features were extracted from different organs (namely, lungs, heart, bladder, stomach, spleen, liver, kidneys) to evaluate changes over time of [^64^Cu] chelator biodistribution. Unlike qualitative analysis, where PET images are visually interpreted, radiomics analyses identify a large number of quantitative PET parameters linked to measurable aspects of the physiological behavior of human or animal bodies. On the other hand, the radiomics feature-extraction process is strongly linked to the target region (i.e., organ) delineation. When target segmentation is performed manually, this process becomes user-dependent. Repeatable results can be obtained by radiomics studies only if the segmentation process is automatic or semi-automatic [12,13]. Therefore, the organ delineation process is crucial to avoid distortions during the feature-extraction task [14]. Furthermore, while conventional segmentation methods can be used for the automatic delineation of tumors in animals, such as in the studies reported in [15], automatic organ segmentation is a prohibitive task due to the impossibility of recognizing the various organs on our CT images (see Figure 1). Consequently, the only way to identify the organs is through the use of an atlas. In our study, all PET images were aligned with the 3D whole-body Digimouse atlas [16]. Starting from this co-registration, radiomics features were extracted from each organ of interest, and statistical analyses were conducted to evaluate the [^64^Cu] chelator biodistribution at different time points after injection.

The remainder of this article has the following structure. Section 2 presents the image dataset, applied data-preparing operations, the proposed radiomics workflow, and the adopted evaluation metrics. Section 3 presents the results and related discussions, and Section 4 concludes the manuscript and indicates future directions for research.

## 2. Materials and Methods

### 2.1. ^64^Cu Chelator and Labeling

**Figure 2:**
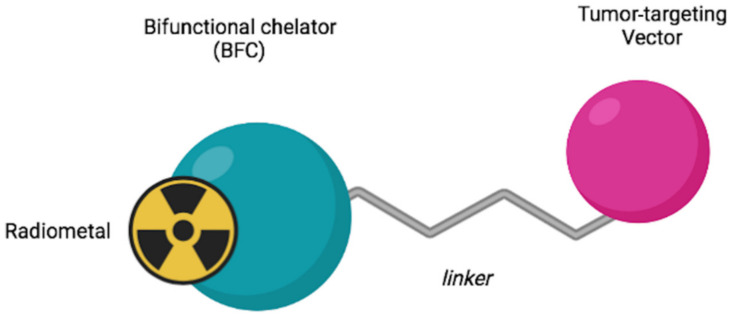
Structure of a radiopharmaceutical having a bifunctional chelator (BFC) incorporated within it.

Radiopharmaceutical compounds generally consist of four main parts: (1) a transport molecule, (2) a linker, (3) a bifunctional chelator, and (4) a metal radionuclide. The chelating fraction acts as a trapping site for the radionuclide, and as an anchor for biomolecules (Figure 2). In our biodistribution study, the component labeled with ^64^Cu is represented only by the chelator. In vivo testing of the whole molecule will be planned in subsequent experiments.

**Figure 3:**
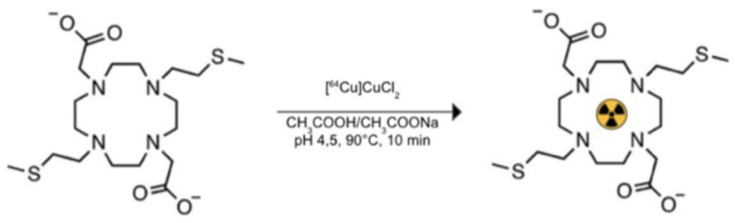
Labeling reaction through ^64^Cu.

In particular, 1,7-bis(2-(methylsulfanyl)ethyl)-4,10,diacetic acid-1,4,7,10-tetraazacyclododecane (DO2A2S), a sulfur-containing derivative of tetraazacyclododecane (cyclen), described in the work conducted by Tosato et al. [9] and synthesized in the context of the “ISOLPHARM” project devoted to producing novel radiopharmaceuticals, was used to chelate ^64^Cu in our preliminary preclinical study of new chelating-radiometal complexes biodistribution on a murine model. DO2A2S was coupled with ^64^Cu to achieve high radiolabeling yield (>98%), and the labeling was carried out at the Cannizzaro Hospital (Catania, Italy). The complexation reaction required harsh reaction conditions, such as acidic pH (about 4.5) and temperature of 90 °C, to be fast (10 min), as shown in Figure 3.

### 2.2. Dataset

Nine 6-week-old female Balb/C nude (CAnN.Cg-Foxn1nu/Crl-Charles River/Envigo)-strain mice weighing 18.0 ± 0.7 g were used. Animals were randomly housed in IVC cages, using a stocking density of 3 mice per cage in individual IVC cages at a constant temperature (23–25 °C) with ad libitum access to food and water. All animals were scanned on Albira Si micro-PET/CT for the in vivo biodistribution analysis of [^64^Cu] DO2A2S at the three time points after injection. A standard diet was given to the mice in the days leading up to the experiment. On the day of injection of the [^64^Cu] chelator, the animals were anesthetized with isoflurane (4% for induction, 2% for maintenance) and the ^64^Cu complex was injected intravenously in the tail with an activity of about ≅7 MBq in a maximum volume of 50 µL. The PET acquisition was performed in three pre-established times (1 h, 4 h, and 24 h), and the main vital parameters were constantly monitored. Mice were divided into three groups: group 1 (micro-PET/CT acquisition after 1 h from the administration of the ^64^Cu complex, n = 3), group 2 (micro-PET/CT acquisition after 4 h from the administration of the ^64^Cu complex, n = 3), and group 3 (micro-PET/CT acquisition after 24 h from the administration of the ^64^Cu complex, n = 3). No suffering occurred after the radiotracer injection procedure. In addition to the PET scan, a morphological diagnostic test (CT) was performed which, through a co-registration with PET images, made it possible to associate the metabolic information with the organ to be studied. The mice were sacrificed by cervical dislocation at the end of the planned follow-up. The organs were preserved in paraffin for subsequent analyses that will not be discussed in this article. All the experiments were carried out after approval by the Italian Ministry of Health (decree: No. 44/2021-PR), and were conducted in accordance with the principle of the Basel Declaration as well as with European and Italian regulations (European Union Directive 2010/63/EU, and Legislative Decree no. 26/2014). All efforts have been made to replace, reduce, and refine the use of laboratory animals. The study also was conducted in accordance with the recommendations of the local committee for animal welfare (OPBA, University of Catania, Catania, Italy); the protocol was approved by the OPBA and the Italian Ministry of Health.

### 2.3. PET/CT Image Acquisition

Micro-PET/CT datasets were acquired using a preclinical micro-PET/CT (Albira Si, Bruker), made available by the CAPiR laboratory (Center for Advanced Preclinical Research in vivo), University of Catania, Italy. The CT datasets were acquired using 600 views in a low-resolution configuration, an initial horizontal position of 37 mm, an FOV of 64 mm, X-ray energy of 35 kV, a current of 200 μA, and the size of each CT-voxel was equal to 500 × 500 × 500 μm^3^. The DICOM (Digital Image Communications in Medicine) images were obtained using a 3D-based FBP algorithm. The PET images were re-constructed using the 3D-MLEM algorithm with a total of 12 iterations. PET-voxel was equal to 500 × 500 × 500 μm^3^.

### 2.4. Atlas Co-Registration

**Figure 4:**
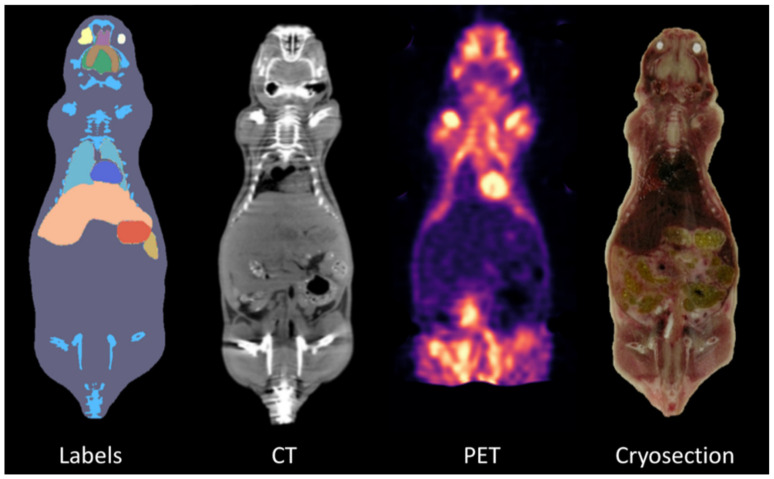
Digimouse Atlas [16].

All PET/CT scans were spatially pre-processed and segmented through co-registration with a standard template space as follows. First, a custom-made MATLAB^®^ [17] algorithm was implemented to rescale the CT image intensity range (from the Hounsfield scale to an 8-bit grayscale format), as well as to segment and remove every non-mouse-related structure from the CT scans, such as the animal holder. After image cleaning, a three-step registration to overlap the 3D whole-body Digimouse atlas [16] with CT scans was performed. This template was assessed to be more appropriate for this study due to the similarity of the anatomical mouse model used, as well as the imaging modalities from which it was constructed (i.e., PET, X-ray CT, and cryosection images of normal nude mice). Despite the multimodal template, in our co-registration pipeline, only the CT part of the atlas was used as its functional part relates to the uptake of a mixture of FDG and ^18^F¯ and therefore the involvement of such information implies an a priori biodistribution pattern, which may alter our results. Figure 4 shows representative coronal sections of the Digimouse atlas [16], together with the organ segmentation labels.

The registration procedure consisted of a semi-automated linear alignment, performed through ITK-Snap software [18], followed by an automated non-linear warping, accomplished with the Elastix toolkit [19], which was further enhanced by small local refinements, located by visual inspection and achieved by the landmark registration tool of 3DSlicer [20]. More in detail, for each mouse, the atlas was first manually pre-aligned to match the subject-specific coordinate system, by means of a rigid roto-translation, so to ease the convergence of the subsequent automated algorithm. Then, the optimal affine transform employing mutual information as the similarity metric with a multi-resolution approach was estimated, involving only half-resolution at the coarsest level and full resolution at the finest because of the poor raw data quality. Afterwards, the non-linear intensity-based registration step was performed by a B-spline deformation model, whose metric, optimization routine, and other parameter settings were chosen as in [21]. This procedure enabled proper overlapping of the global mouse shape outline and it considerably improved the registration of the main anatomical structures with the highest contrast, such as the spine, skull, and limbs; nevertheless, the expected misalignments in highly low-contrast tissues of interest, such as the bladder, as well as some slight residual differences in the lung contour, required some local refinements; thus, a thin-plate spline mapping through multiple landmark definition, where the fiducial markers were manually positioned by visual inspection, was generated. Finally, all these estimated linear and non-linear transforms were exploited to warp the binary masks of the selected regions of interest (ROIs) into each subject-specific space.

### 2.5. Extraction of Radiomics Features

After atlas co-registration, organs of interest (i.e., heart, bladder, stomach, spleen, liver, kidneys, and lungs) were identified and exported as binary masks (background 0, organ of interest 1). Before proceeding to the feature-extraction process, the PET DICOMs were modified to incorporate the standardized uptake value (SUV) as reported in [22,23]. The SUV is the most common semi-quantitative parameter used to estimate biodistribution in PET images. The SUV normalizes the voxel activity considering acquisition time, administered activity, and mouse weight. In other words, PET images were converted to SUV images. In this way, PET images took into account factors that would otherwise be ignored during radiomics analysis. At this point, both PET images and co-registered masks were used to extract 108 radiomics features using an Image Biomarker Standardization Initiative (IBSI) [24]-compliant analysis software, namely, PyRadiomics [25], since one of the main points in radiomics studies is to increase the reproducibility of the extracted features [26]. In this way, through a large panel of engineered hard-coded algorithms, reproducible radiomics features can be obtained. PyRadiomics is implemented in Python, a language that has established itself as a popular open-source language for scientific computing, and can be installed on any system. PyRadiomics provides a flexible analysis platform with a back-end interface allowing automation in data processing, feature definition, and batch handling. This software extracts different feature classes, namely, shape descriptors, first-order statistics, and the following texture matrices: gray-level co-occurrence matrix (GLCM), gray-level run-length matrix (GLRLM), gray-level dependence matrix (GLDM), gray-level size-zone matrix (GLSZM), and neighboring gray-level dependence matrix (NGLDM).

Shape descriptors are independent of the gray-level intensity distribution in the image. They describe geometric aspects such as volume, maximum diameter, surface area, compactness, and sphericity. In detail, the surface area is calculated through a process that produces a net of triangles that completely cover the organ surface (namely, triangulation) and is used to calculate the surface–volume ratio, while compactness and sphericity describe how the organ shape (from a functional point of view) differs from that of a circle (2D) or a sphere (3D).

First-order statistical descriptors (or “histogram-based” features) describe the frequency distribution of voxels within an organ through the analysis of the histogram of gray-level intensity values. Among others are skewness which reflects the asymmetry of the data distribution curve to lower (negative) or higher (positive) values than the mean, kurtosis, which reflects the tail of data distribution with respect to a gaussian distribution due to outliers, entropy, and uniformity.

Finally, texture features are used to evaluate the relative voxel positions within the image, providing information on the spatial organization of gray-levels in the organ of interest. These features are classified according to the following texture classes from which they are obtained: (i) GLCM quantifies the incidence of voxels with the same intensities at a predetermined distance along a fixed direction, (ii) GLRLM quantifies consecutive voxels with the same intensity along fixed directions, (iii) GLDM quantifies the number of voxel segments having the same intensity in a given direction, (iv) GLSZM quantifies the number of connected voxels that have equal gray-level intensity, and (v) NGTDM quantifies the spatial interrelationships between three or more voxels.

### 2.6. Statistical Analyses

The performance result of the proposed approach was obtained using the one-way analysis of variance (ANOVA) and post hoc Tukey Honestly Significant Difference (HSD) test. One-way ANOVA is used to determine whether there are any statistically significant differences between the means of three or more independent groups by comparing the variability within these groups with the variability between groups. In other words, the one-way ANOVA compares the means among groups (in our case the three different time points after the injection of the ^64^Cu-labeled chelator), determining if one of these means is statistically different from the others.

Specifically, it tests the null hypothesis:

(1) $$H_{0}=\mu_{1}=\mu_{2}\;\cdots \;\mu_{k}$$

where *µ* = mean group, and *k* = number of groups. If the one-way ANOVA returns a statistically significant result (*p*-value lower than 0.05), we accept the alternative hypothesis: there are at least two group means that are statistically significantly different from each other. Then, we considered the radiomics features with *p*-value < 0.05 in one-way ANOVA to find the percentage of radiomics features (out of 108 overall radiomics features) different between at least two groups. However, ANOVA does not give information on which group is significantly different from the others, only that at least two groups are statistically different. For this reason, the Tukey HSD test must be used. This is a post hoc test based on the Studentized range distribution useful for identifying which of the group pairs are significantly different from each other. A parameter for each pair is calculated, namely, Tukey HSD Q-statistic, as:

(2) $$Q-\mathrm{statistic}\;=\frac{{\bar{X}}_{i}-{\bar{X}}_{j}}{\sqrt{s_{w}^{2}/n}\;}$$

where ${\bar{X}}_{i}$ and ${\bar{X}}_{j}$ are the means of the compared samples, n is the size of the sample, and $s_{w}^{2}$ is the within-group variance. Then, the *p*-value of the comparison between observed Q-statistic and Q-critical (this value can be found in the tables of the inverse Studentized range distribution [27]) was calculated. Finally, radiomics features with Tukey HSD *p*-value < 0.05 were considered to identify the percentage of radiomics features (out of 108 overall radiomics features) different between each of the three pairs of groups to identify which of them showed a statistically significant difference.

## 3. Results and Discussions

**Figure 5:**
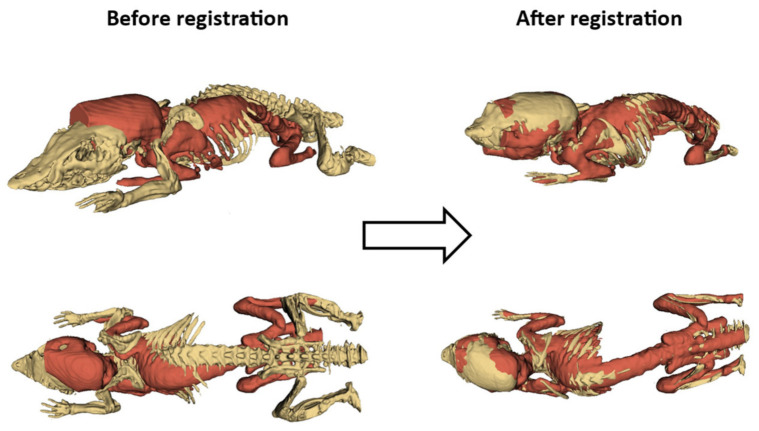
Qualitative registration performance, assessed through the overlapping of standard (yellow) and real (red) bone structures.

Figure 5 shows the 3D segmented skeleton of the Digimouse atlas overlapped with a representative mouse, both before and after the registration procedure; quantitatively, this workflow reached registration accuracies ranging from 87.5% to 91%, with a mean performance of 89% ± 1%, in terms of intensity correlation between the warped atlas and the original CT images.

After atlas co-registration, radiomics features from PET images were extracted from seven different targets: heart, bladder, stomach, liver, spleen, kidney, and lung.

**Figure 6:**
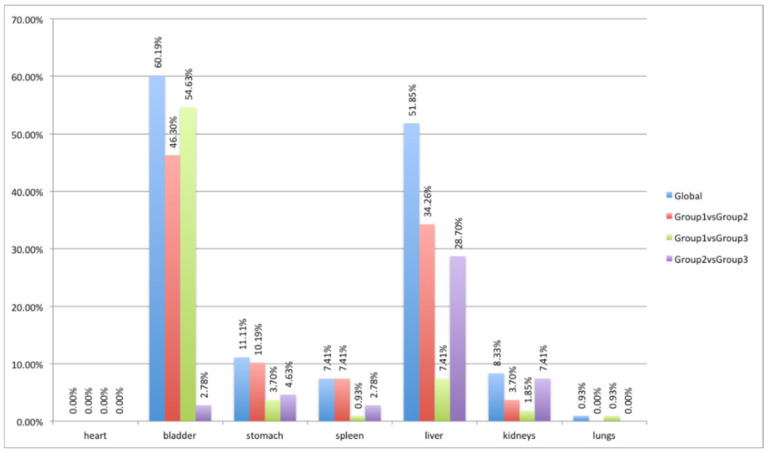
ANOVA and HSD test analyses showing differences in the extracted radiomics features in the three mouse groups (blue column) and differences considering pairs of groups: first group versus second group (red column), first group versus third group (green column), and second group versus third group (violet column).

ANOVA and HSD analyses showed different behavior according to the investigated organ at the three different time points after the injection of the ^64^Cu-labeled chelator, as shown in Figure 6 and Table 1. Specifically, in the heart and lung districts, less than 0.93% of 108 features extracted showed significant variations (*p*-value lower than 0.05) among the three groups of mice. This was a predictable result as uptake in these organs is very low (SUV_max_ and SUV_mean_ were 0.36 ± 0.98 and 0.12 ± 0.02 in the heart, 0.43 ± 0.09 and 0.13 ± 0.02 in the lung). An intermediate situation occurred in the stomach, spleen, and kidney districts, where a maximum of 11.11% features were statistically different. Additionally, in this case, the PET uptake was quite low in all three groups (SUV_max_ and SUV_mean_ were 0.53 ± 0.15 and 0.26 ± 0.11 in the stomach, 0.36 ± 0.11 and 0.16 ± 0.05 in the spleen, and 0.51 ± 0.16 and 0.20 ± 0.06 in the kidney). In the stomach and kidney districts, the greatest differences occurred between group 1 and group 2, while in the kidney district the greatest differences occurred between group 2 and group 3. Finally, over 60% and 51% of features were different in the bladder and liver districts, respectively. In these districts, the uptake was much higher than in the other districts (SUV_max_ and SUV_mean_ were 4.54 ± 6.64 and 2.25 ± 3.34 in the bladder, and 0.86 ± 0.24 and 0.35 ± 0.08 in the liver), and large variations occurred between group 1 and group 2, and group 1 and group 3 for bladder, and between group 1 and group 2, and group 2 and group 3 for the liver.

**Figure 7:**
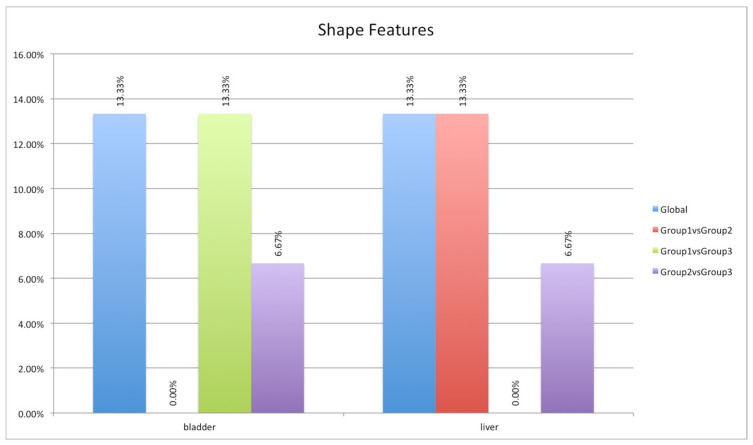
ANOVA and HSD test analyses showing differences in the shape feature group (15 features) in the three mouse groups (blue column) and differences considering pairs of groups: first group versus second group (red column), first group versus third group (green column), and second group versus third group (violet column).

Focusing on the two districts (i.e., bladder and liver) where most of the features showed significant variations among the three groups of mice, a particular analysis was performed considering the features divided into three feature classes (see Section 2.5): shape (15 features), first-order (18 features), and texture (75 features). The least-susceptible class to acquisition time was the shape, as expected. This feature class concerns geometric aspects of the target of interest such as volume, maximum diameter, surface area, compactness, and sphericity. Obviously, it does not concern the shape from the morphological point of view (as in the CT images) but from the point of view of the [^64^Cu] chelator biodistribution. Therefore, a minimal difference was to be expected (see Figure 7): two features (namely, shape_Flatness and shape_LeastAxisLength) on 15 features showed a significant variation in the three groups.

**Figure 8:**
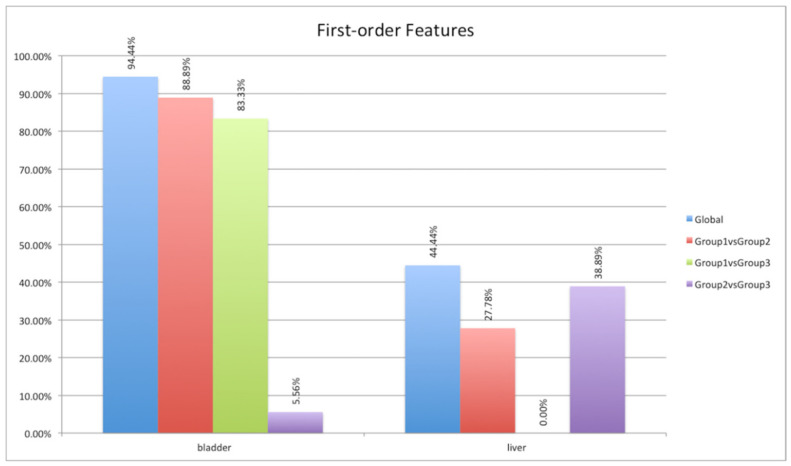
ANOVA and HSD test analyses showing differences in the first-order feature group (18 features) in the three mouse groups (blue column) and differences considering pairs of groups: first group versus second group (red column), first group versus third group (green column), and second group versus third group (violet column).

In the same way, the first-order class was analyzed (see Figure 8). This class concerns “histogram-based” features describing the frequency distribution of voxels within the target through the analysis of the histogram of gray-level intensity values. Contrary to the first class examined, most of the features belonging to this group varied significantly in the bladder district considering group 1 compared to other groups. This was due to the fact that after one hour (mice of the first group) the chelator had not been eliminated from the body. A much lower ^64^Cu concentration occurred at 4 and 24 h. Regarding the liver district, more than 44% of the extracted features varied over time. Quite unexpectedly, there was no variation between the first group and the third group. More in-depth investigations will be needed to analyze this aspect.

**Figure 9:**
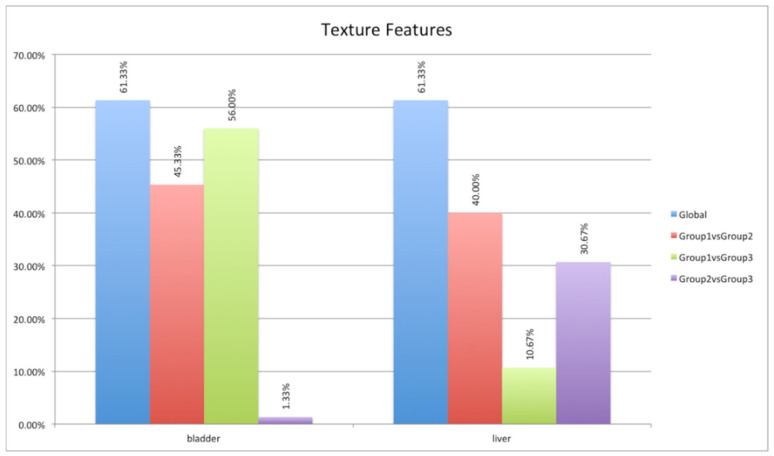
ANOVA and HSD test analyses showing differences in the texture group (75 features) in the three mouse groups (blue column) and differences considering pairs of groups: first group versus second group (red column), first group versus third group (green column), and second group versus third group (violet column).

Concerning the texture class, like the previous class, this one also strongly depends on the gray levels and therefore on the [^64^Cu] chelator biodistribution. Especially, it provides information on the spatial organization of gray levels in the organ of interest. For both districts, more than 61% of the extracted features varied over time (see Figure 9), obtaining results similar to the previous analysis (minimal difference between group 2 and 3 in the bladder district, and between group 1 and 3 in the liver district).

**Figure 10:**
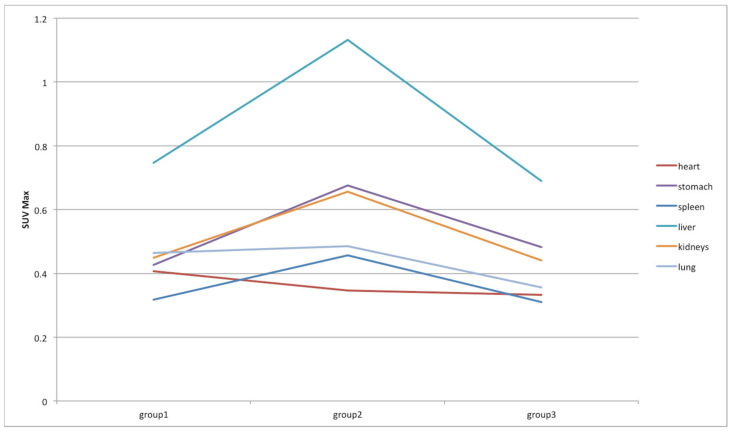
SUV_max_ variations among groups in heart, stomach, spleen, liver, kidneys, and lung districts.

**Figure 11:**
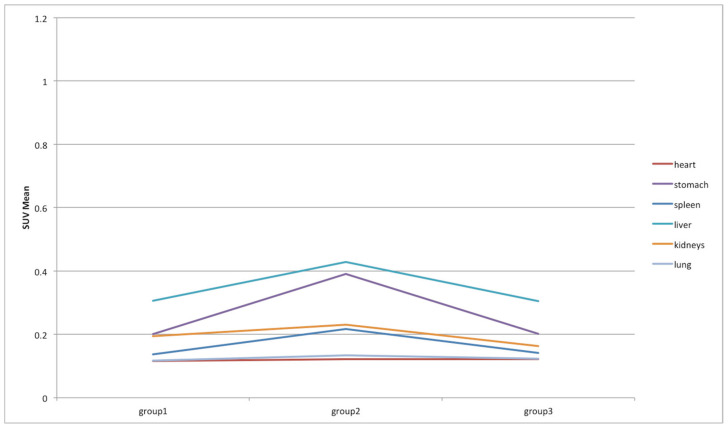
SUV_mean_ variations among groups in heart, stomach, spleen, liver, kidneys, and lung districts.

**Figure 12:**
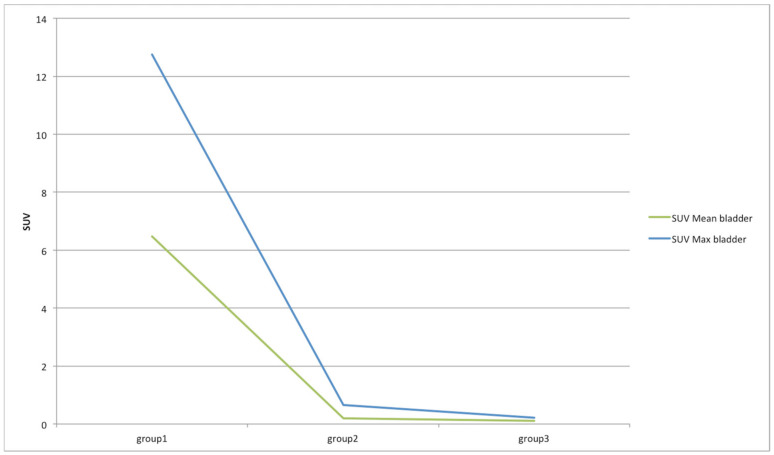
SUV_max_ and SUV_mean_ variations among groups in the bladder districts.

Finally, a traditional SUV-based analysis was performed to assess its variations over time. Specifically, SUV_max_ and SUV_mean_ were considered and their variations in the different organs are shown in Figure 10 and Figure 11, respectively. For easier reading, we excluded in Figure 10 and Figure 11 the bladder, whose SUVs were higher than the other organs, making them unreadable. Therefore, the SUV variations in the bladder are shown in Figure 12. As expected, the SUV_max_ is susceptible to greater variation between groups but this is also due to the noise [28]. As a matter of fact, the SUV_max_ corresponds to the maximum voxel value in the target, and a single voxel may not be representative of the overall target uptake. This value is most resistant to partial volume effect (PVE) and is operator-independent, but it is highly variable due to the high noise level in PET data [29]. Consequently, it is advisable to also focus on the results of the SUV_mean_, which is more stable and whose behavior is in line with the results obtained from the previous analysis based on the proposed decision support system. Specifically, liver, bladder, and stomach showed the highest variations in terms of SUV_mean_. Similarly, in the analysis based on the proposed decision support system, SUV_max_ and SUV_mean_ were among the features that showed significant variations in these three organs. This has not happened in any other organ. Notably, SUV_max_ varied significantly between group 1 and 2, and group 1 and 3 in the bladder, and between group 1 and 2, and group 2 and 3 in the liver. SUV_mean_ varied significantly between group 2 and 3 in the bladder, between group 2 and 3 in the stomach, and between group 2 and 3 in the liver.

## 4. Conclusions

In this study, we investigated a semi-automatic strategy, satisfying the increasing need to obtain repeatable results in biomedical imaging studies [30,31], to identify several mouse organs, and to investigate, through radiomics analyses, possible changes over time in [^64^Cu] chelator biodistribution using micro-PET imaging. The automatic or semi-automatic identification of the volume of interest is the first step of a radiomics analysis since, if the target identification is performed manually, extracted features are characterized by high inter-observer variability [32]. Although deep learning methods are more efficient than classical statistical approaches, obtaining better performance in image segmentation of several anatomic districts, such as in [33,34] where our group developed a deep learning algorithm for biomedical images that requires limited hardware resource benefiting from reduced training data requirements, in this study, it was not applicable due to the poor resolution of the CT, where mouse organs were not easily recognizable. For this reason, an alternative solution based on atlas co-registration was preferred. Our results demonstrated the feasibility and efficacy of our approach, obtaining a good co-registration accuracy in terms of intensity correlation between the warped atlas and the CT images. After this step, atlas masks were reported in PET images to automatically extract 108 features from different organs, namely, heart, bladder, stomach, spleen, liver, kidneys, and lungs. In this way, the [^64^Cu] chelator biodistribution over time in micro-PET examinations were analyzed. Specifically, three-time points after radiotracer injection in mice that underwent micro-PET/CT imaging were considered. The nine mice were divided into three groups of equal size (n = 3); each group underwent micro-PET at a different acquisition time (1 h, 4 h, 24 h after radiotracer administration, respectively). A very low percentage of radiomics features showed significant variations (*p*-value < 0.05) between the three groups of mice in organs such as heart, stomach, spleen, kidney, and lungs as shown in statistical analyses. This was an expected result as the accumulation of ^64^Cu activity in these organs remained low. Vice versa, in liver and bladder districts, the biodistribution of [^64^Cu] chelator was high, and high quantitative differences were noted in the three different acquisition times.

The lack of a correction method for the precise identification of small tissues should be considered as a major limitation of this study: the PVE is the most important factor impacting the quality and the quantitative accuracy in PET studies. The under-estimation of PET activity due to the PVE cannot be assumed to be negligible [28]. The separation of target (in our study, the different organs) and background voxels is difficult. A PVE correction method should be used during DICOM-SUV conversion, as described in Section 2.5, to improve quantitative measures [35].

In conclusion, this preliminary study is fundamental for subsequent preclinical studies as an easily adaptable workflow has been implemented for future preclinical experiments. Therefore, our innovative system may improve the method of observing the biodistribution of other radiolabeled chelators, and open the way towards a decision support system in the context of new radiopharmaceutical studies that will be conducted through preclinical experiments using PET imaging. Furthermore, the analysis of the radiopharmaceutical uptake curve from 0 to 4 h would be interesting to analyze with our innovative tool in future studies.

## Figures and Tables

**Table 1:** ANOVA and HSD test analyses based on the seven districts investigated. In each row is reported the percentage of radiomics features that showed significant variations (*p*-value < 0.05) between the three groups of mice.

	Global	Group 1 vs. Group 2	Group 1 vs. Group 3	Group 2 vs. Group 3
Heart	0.00%	0.00%	0.00%	0.00%
Bladder	60.19%	46.30%	54.63%	2.78%
Stomach	11.11%	10.19%	3.70%	4.63%
Spleen	7.41%	7.41%	0.93%	2.78%
Liver	51.85%	34.26%	7.41%	28.70%
Kidney	8.33%	3.70%	1.85%	7.41%
Lung	0.93%	0.00%	0.93%	0.00%

## Data Availability

Data are available for bona fide researchers who request it from the authors.

## References

[1] Talmadge J.E., Singh R.K., Fidler I.J., Raz A. (2007). Murine models to evaluate novel and conventional therapeutic strategies for cancer. Am. J. Pathol..

[2] Minn A.J., Gupta G.P., Siegel P.M., Bos P.D., Shu W., Giri D.D., Viale A., Olshen A.B., Gerald W.L., Massagué J. (2005). Genes that mediate breast cancer metastasis to lung. Nature.

[3] An Z., Jiang P., Wang X., Moossa A.R., Hoffman R.M. (1999). Development of a high metastatic orthotopic model of human renal cell carcinoma in nude mice: Benefits of fragment implantation compared to cell-suspension injection. Clin. Exp. Metastasis.

[4] Hoffman R.M. (1999). Orthotopic metastatic mouse models for anticancer drug discovery and evaluation: A bridge to the clinic. Investig. New Drugs.

[5] Okoye N.C., Baumeister J.E., Khosroshahi F.N., Hennkens H.M., Jurisson S.S. (2019). Chelators and metal complex stability for radiopharmaceutical applications. Radiochim. Acta.

[6] Zhou Y., Li J., Xu X., Zhao M., Zhang B., Deng S., Wu Y. (2019). ^64^Cu-based Radiopharmaceuticals in Molecular Imaging. Technol. Cancer Res. Treat..

[7] Niccoli Asabella A., Cascini G.L., Altini C., Paparella D., Notaristefano A., Rubini G. (2014). The copper radioisotopes: A systematic review with special interest to ^64^Cu. Biomed Res. Int..

[8] Follacchio G.A., De Feo M.S., De Vincentis G., Monteleone F., Liberatore M. (2018). Radiopharmaceuticals Labelled with Copper Radionuclides: Clinical Results in Human Beings. Curr. Radiopharm..

[9] Tosato M., Dalla Tiezza M., May N.V., Isse A.A., Nardella S., Orian L., Verona M., Vaccarin C., Alker A., MäcKe H. (2021). Copper Coordination Chemistry of Sulfur Pendant Cyclen Derivatives: An Attempt to Hinder the Reductive-Induced Demetalation in 64/67Cu Radiopharmaceuticals. Inorg. Chem..

[10] Anderson C.J., Ferdani R. (2009). Copper-64 radiopharmaceuticals for PET imaging of cancer: Advances in preclinical and clinical research. Cancer Biother. Radiopharm..

[11] Laudicella R., Comelli A., Liberini V., Vento A., Stefano A., Spataro A., Crocè L., Baldari S., Bambaci M., Deandreis D. (2022). [^68^Ga]DOTATOC PET/CT Radiomics to Predict the Response in GEP-NETs Undergoing [^177^Lu]DOTATOC PRRT: The “Theragnomics” Concept. Cancers.

[12] Barone S., Cannella R., Comelli A., Pellegrino A., Salvaggio G., Stefano A., Vernuccio F. (2021). Hybrid descriptive-inferential method for key feature selection in prostate cancer radiomics. Appl. Stoch. Model. Bus. Ind..

[13] Stanzione A., Ponsiglione A., Di Fiore G.A., Picchi S.G., Di Stasi M., Verde F., Petretta M., Imbriaco M., Cuocolo R. (2020). Prostate Volume Estimation on MRI: Accuracy and Effects of Ellipsoid and Bullet-Shaped Measurements on PSA Density. Acad. Radiol..

[14] Alongi P., Stefano A., Comelli A., Laudicella R., Scalisi S., Arnone G., Barone S., Spada M., Purpura P., Bartolotta T.V. (2021). Radiomics analysis of 18F-Choline PET/CT in the prediction of disease outcome in high-risk prostate cancer: An explorative study on machine learning feature classification in 94 patients. Eur. Radiol..

[15] Raccagni I., Belloli S., Valtorta S., Stefano A., Presotto L., Pascali C., Bogni A., Tortoreto M., Zaffaroni N., Daidone M.G. (2018). [^18^F]FDG and [^18^F]FLT PET for the evaluation of response to neo-adjuvant chemotherapy in a model of triple negative breast cancer. PLoS ONE.

[16] Dogdas B., Stout D., Chatziioannou A.F., Leahy R.M. (2007). Digimouse: A 3D whole body mouse atlas from CT and cryosection data. Phys. Med. Biol..

[17] Sharma G., Martin J. (2009). MATLAB^®^: A language for parallel computing. Int. J. Parallel Program..

[18] Yushkevich P.A., Gao Y., Gerig G. ITK-SNAP: An interactive tool for semi-automatic segmentation of multi-modality biomedical images. Proceedings of the 2016 38th Annual International Conference of the IEEE Engineering in Medicine and Biology Society (EMBC).

[19] Klein S., Staring M., Murphy K., Viergever M.A., Pluim J.P.W. (2010). elastix: A toolbox for intensity-based medical image registration. IEEE Trans. Med. Imaging.

[20] Fedorov A., Beichel R., Kalpathy-Cramer J., Finet J., Fillion-Robin J.C., Pujol S., Bauer C., Jennings D., Fennessy F., Sonka M. (2012). 3D Slicer as an Image Computing Platform for the Quantitative Imaging Network. Magn. Reson. Imaging.

[21] Baiker M., Staring M., Löwik C.W.G.M., Reiber J.H.C., Lelieveldt B.P.F. (2011). Automated registration of whole-body follow-up MicroCT data of mice. Med. Image Comput. Comput. Assist. Interv..

[22] Stefano A., Vitabile S., Russo G., Ippolito M., Marletta F., D’Arrigo C., D’Urso D., Gambino O., Pirrone R., Ardizzone E. (2016). A fully automatic method for biological target volume segmentation of brain metastases. Int. J. Imaging Syst. Technol..

[23] Comelli A., Stefano A., Benfante V., Russo G. (2018). Normal and Abnormal Tissue Classification in Positron Emission Tomography Oncological Studies. Pattern Recognit. Image Anal..

[24] Fornacon-Wood I., Mistry H., Ackermann C.J., Blackhall F., McPartlin A., Faivre-Finn C., Price G.J., O’Connor J.P.B. (2020). Reliability and prognostic value of radiomic features are highly dependent on choice of feature extraction platform. Eur. Radiol..

[25] Van Griethuysen J.J.M., Fedorov A., Parmar C., Hosny A., Aucoin N., Narayan V., Beets-Tan R.G.H., Fillion-Robin J.C., Pieper S., Aerts H.J.W.L. (2017). Computational radiomics system to decode the radiographic phenotype. Cancer Res..

[26] Stefano A., Leal A., Richiusa S., Trang P., Comelli A., Benfante V., Cosentino S., Sabini M.G., Tuttolomondo A., Altieri R. (2021). Robustness of pet radiomics features: Impact of co-registration with mri. Appl. Sci..

[27] Kokoska S., Nevison C. (1989). Critical Values For The Studentized Range Distribution. Statistical Tables and Formulae.

[28] Soret M., Bacharach S.L., Buvat I.I. (2007). Partial-volume effect in PET tumor imaging. J. Nucl. Med..

[29] Banna G.L., Anile G., Russo G., Vigneri P., Castaing M., Nicolosi M., Strano S., Gieri S., Spina R., Patanè D. (2017). Predictive and Prognostic Value of Early Disease Progression by PET Evaluation in Advanced Non-Small Cell Lung Cancer. Oncology.

[30] Stefano A., Gioè M., Russo G., Palmucci S., Torrisi S.E., Bignardi S., Basile A., Comelli A., Benfante V., Sambataro G. (2020). Performance of Radiomics Features in the Quantification of Idiopathic Pulmonary Fibrosis from HRCT. Diagnostics.

[31] Cuocolo R., Stanzione A., Ponsiglione A., Romeo V., Verde F., Creta M., La Rocca R., Longo N., Pace L., Imbriaco M. (2019). Clinically significant prostate cancer detection on MRI: A radiomic shape features study. Eur. J. Radiol..

[32] Comelli A., Stefano A., Coronnello C., Russo G., Vernuccio F., Cannella R., Salvaggio G., Lagalla R., Barone S. (2020). Radiomics: A New Biomedical Workflow to Create a Predictive Model. Communications in Computer and Information Science.

[33] Stefano A., Comelli A. (2021). Customized efficient neural network for covid-19 infected region identification in ct images. J. Imaging.

[34] Cuocolo R., Comelli A., Stefano A., Benfante V., Dahiya N., Stanzione A., Castaldo A., De Lucia D.R., Yezzi A., Imbriaco M. (2021). Deep Learning Whole-Gland and Zonal Prostate Segmentation on a Public MRI Dataset. J. Magn. Reson. Imaging.

[35] Stefano A., Gallivanone F., Messa C.L., Gilardi M.C.L., Castiglioni I. (2014). Metabolic impact of Partial Volume Correction of [^18^F]FDG PET-CT oncological studies on the assessment of tumor response to treatment. Q. J. Nucl. Med. Mol. Imaging.

